# Localizing Movement-Related Primary Sensorimotor Cortices with Multi-Band EEG Frequency Changes and Functional MRI

**DOI:** 10.1371/journal.pone.0112103

**Published:** 2014-11-06

**Authors:** Ching-Chang Kuo, Phan Luu, Kyle K. Morgan, Mark Dow, Colin Davey, Jasmine Song, Allen D. Malony, Don M. Tucker

**Affiliations:** 1 Electrical Geodesics, Inc., Eugene, Oregon, United States of America; 2 NeuroInformatics Center, University of Oregon, Eugene, Oregon, United States of America; 3 Department of Psychology, University of Oregon, Eugene, Oregon, United States of America; 4 Department of Computer and Information Science, University of Oregon, Eugene, Oregon, United States of America; ARC Centre of Excellence in Cognition and its Disorders (CCD), Australia

## Abstract

Electroencephalographic (EEG) oscillations in multiple frequency bands can be observed during functional activity of the cerebral cortex. An important question is whether activity of focal areas of cortex, such as during finger movements, is tracked by focal oscillatory EEG changes. Although a number of studies have compared EEG changes to functional MRI hemodynamic responses, we can find no previous research that relates the fMRI hemodynamic activity to localization of the multiple EEG frequency changes observed in motor tasks. In the present study, five participants performed similar thumb and finger movement tasks in parallel EEG and functional MRI studies. We examined changes in five frequency bands (from 5–120 Hz) and localized them using 256 dense-array EEG (dEEG) recordings and high-resolution individual head models. These localizations were compared with fMRI localizations in the same participants. Results showed that beta-band (14–30 Hz) desynchronizations (power decreases) were the most robust effects, appearing in all individuals, consistently localized to the hand region of the primary motor cortex, and consistently aligned with fMRI localizations.

## Introduction

The synchronization and desynchronization of oscillatory electroencephalographic (EEG) activity is believed to reflect basic neurophysiological processes that are fundamental to information processing by the brain. Oscillatory events provide clues to how information is organized within particular brain regions, and how it is shared between multiple regions to form functional networks [1], [2], [3], [4], [5]. For example, previous studies have shown that increases and decreases in beta-band oscillations (13 to 30 Hz) and high gamma-band activities (over 60 Hz) play important roles in motor control [6], [7].

In invasive studies, local field potentials recorded from intracranial electrodes over sensorimotor cortex revealed rhythmic neural activities that were interrupted by activating neurons involved in movement preparation and execution [8]. Using noninvasive EEG and MEG techniques, researchers have shown frequency specific amplitude (or spectral power) changes before and after movements. These changes involve decreases in amplitude, perhaps reflecting the interruption of ongoing oscillatory activity by functionally active neuronal populations, as shown by Murthy and Fetz [8], or increases relative to a specified baseline. Decreases are referred to as event-related desynchronization (ERD), and increases are referred to as event-related synchronization (ERS) [9].

ERDs in the beta (13–30 Hz) band are observed approximately 500 ms before a movement, and they can last until movement execution [10]. A corresponding rebound or ERS in the beta band is typically observed 300 to 1000 ms after movement onset [11], [12]. The beta ERD/ERS pattern can also be observed during passive movement, motor imagery [13], and action observation [14].

EEG/MEG studies have observed changes in other frequency bands during motor tasks as well [12], [15], [16], [17], [18]. Oscillations in the theta band (4–7 Hz) ERS have been observed during pre-movement, movement, and post-movement intervals [19], [20]. This theta band activity may contribute to event-related potential averages because it becomes phase-locked to the movement [1]. This theta band activity is believed to reflect coordination of premotor and motor activity with response monitoring functions of the medial prefrontal cortex [20].

Mu-band (8–13 Hz, also referred to as alpha-band) ERD is observed during the same time interval as the beta-band ERD activity. It is believed that both mu and beta band activity may reflect the coordination of the motor act with sensory (e.g., movement cues) and cognitive processes [11]. Unlike beta-band activity, mu-band activity does not show a post movement ERS but rather ERD. The post-movement mu-band ERD is believed to reflect the continued activation (perhaps subthreshold) of the motor cortex.

In contrast, the post-movement beta ERS is thought to reflect motor inhibition and the processing of somatosensory feedback, although this interpretation remains controversial [21]. An alternative hypothesis is that the post-movement beta-band ERS might reflect a reset of the motor system in order to prepare for (or a switch to) the next movement [22].

Recent research has revealed changes in the high frequency bands associated with movements, including the low-gamma (LG, 31–60 Hz) and high gamma frequency bands (HG, 61–250 Hz). Like activity at mu and beta bands, HG band activity is also observed in the pre-movement period [16]. However, HG band activity in this interval is seen as a power increase (i.e., ERS). Also like mu and beta band ERDs, the HG band ERS is localized at the scalp to the contralateral electrodes that lay over the motor cortex. HG-band activity appears more focal than either mu or beta ERDs and therefore may reflect local recurrent network processes (e.g., binding of neuronal activity within a small neuronal population) involved in the formation and maintenance of a motor activity [23], [24]. In contrast to HG-band activity, the mu and beta frequency are believed to be too slow to support local processes [25]. Evidence of these HG activities, observed from MEG studies, has been corroborated mainly with EEG recordings using invasive electrocorticogram (ECoG) recordings [26], [27], [28]. We are only aware of a few scalp EEG studies examining motor related HG activity [15], [16], [25]. These studies found movement-related HG activity that was localized to the contralateral motor area.

Given the close relationship of electrophysiological recordings to neuronal activity, an important question has been the relation between these measures and the BOLD hemodynamic response of fMRI. An influential study by Logothetis et al. examined this question with invasive recordings in monkeys [29]. The results showed that local field potentials in the gamma range were positively correlated with the BOLD response; neuronal action potentials were not significantly related to the BOLD response. Since that seminal study, many studies have been performed in humans with intracranial recordings to examine the relation of local EEG oscillations to the BOLD response. As reviewed by Ojemann et al. [30], gamma-band activity, as recorded by intracranial electrodes, is typically observed to be positively correlated with BOLD activation, whereas lower frequency intracranial EEG activity is negatively correlated with BOLD.

The relation between BOLD activity with scalp recorded EEG has been observed as well. For example, Scheeringa and colleagues [17] confirmed that gamma-band activity is indeed positively correlated with the BOLD response and that activity at lower frequencies (i.e., alpha and beta) are negatively correlated. Moreover, gamma- and lower-band activities contribute uniquely to prediction of the BOLD response. However, studies that examined the relation between scalp EEG and hemodynamic response used simultaneous EEG and fMRI recordings, making source localization of the EEG activity unfeasible, due to the sensitivity of source analysis techniques to noise. This shortcoming is a potential limitation when the goal is to understand the relation of EEG oscillations to the BOLD response in specific regions of cortex. Recently, Yaun et al. [31] performed parallel recordings, allowing localization of EEG activity in individual subjects, and compared the source activity to BOLD data. They confirmed that activities in the alpha and beta bands in the sensorimotor cortex are negatively correlated with fMRI activation in the same region.

Given the interest in the relation between BOLD activity and EEG signals and the functional significance of signals in different EEG bands, knowledge about the localization of each signal (and their reliability) relative to the BOLD activations will contribute to our understanding of this relation. However, in order to answer this question and because source estimation of the EEG data is exquisitely sensitive to noise, EEG-fMRI joint recordings are not appropriate due to the difficulty associated with cleaning the ballistocardigram artifact from the EEG when recorded in the MRI scanner. In addition to the strict requirement for artifact free data for accurate source estimates, source estimation also requires accurate head models to capture the tissue geometries and conductive properties of individual participants. These parameters describe how current propagate from the cortex to where they are measured on the scalp, and mischaracterization of these parameters compromise the accuracy of source estimates [4]. Finally, for comparison with BOLD data, the head model used for source estimates should be relevant for the registration of the BOLD data.

In the present study, parallel recordings (rather than joint) were performed in the same participants performing similar motor tasks, and high-resolution head models were constructed for each individual. We employed improved methods of electrical source imaging (ESI) with a 256 dense-array electroencephalographic (dEEG) recording and examined the localization of all the typical frequency bands of the EEG in relation to the locations of the BOLD responses.

## Materials and Methods

### 1. Participants

Five participants with normal or corrected-to-normal vision (3 males and 2 females, age 22–55, all right handed users) were recruited to participate in both EEG and fMRI studies. All participants reported no history of neurological disorders nor were they taking medications that are known to affect the EEG (e.g., anticonvulsants). All participants provided and signed informed written consent prior to participation. They were given a copy as a reference and reminder of the information conveyed. Institutional Review Boards (IRB) at Electrical Geodesics Inc. (EGI) and the University of Oregon approved the human subject use protocol for the present study.

### 2. EEG Acquisition

**Figure 1:**
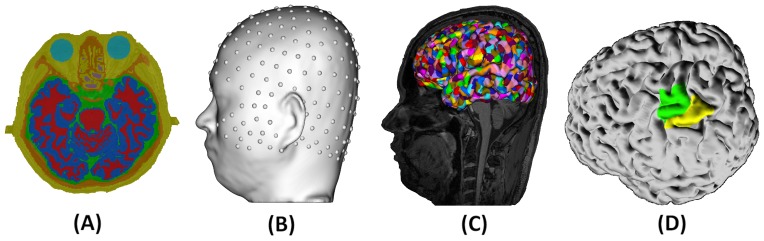
Individual head model for EEG source estimation and BOLD registration. A) Tissue segmentation of scalp (yellow), skull (brown), cerebral-spinal fluid (green), gray matter (blue), white matter (red), and air (purple). B) 256-channel EEG sensors registered on the scalp. C) Reconstructed cortical surface. Colors patches on the cortical surface represent dipole patches. D) M1 (green) and S1 (yellow) ROIs defined for the left hemisphere in one subject.

EEG data were acquired with a 256-channel HydroCel Geodesic Sensor Net (EGI, Eugene, OR) using Net Station 4.5 software. The locations of 256 electrodes are shown in Figure 1(B). All electrode impedances were below 70 KΩ before recording was started [32]. Recordings were referenced to the Cz electrode. The data was digitized with a 24-bit A/D converter at a 1 KHz sample rate.

### 3. Tasks and EEG Recording

Participants were seated 60 cm in front of a monitor with their chin on a chin rest, which standardized positions across all participants and minimized head movements. Participants were instructed to place the response hand (either left or right hand) on a 4-button response pad. A stimulus (the words “Thumb” or “Pinky”) was presented on the screen to inform subjects which finger to use (note that the “Pinky” condition was not analyzed in the present study). The stimulus remained on the screen until it changed to instruct participants to use a different finger. Participants were asked to fixate on the stimulus in order to minimize eye movements.

Prior to data acquisition, participants were provided an opportunity to practice the task to get a feel for the pace of the task, and the experimenter provided participants with feedback about their performance relative to the.5 Hz target rate. That is, although the cue instructed participants which finger to use, it did not instruct them when to move the finger; participants decided when to move the finger with the goal of making a movement once every two seconds. All participants were able to establish a response rate close to the 0.5 Hz target rate after a few minutes of practice.

The study was divided into eight six-minute blocks. Participants were allowed to take short breaks between blocks. Response hand was grouped by block, and within each block the finger used was determined by the cue, alternating between thumb and little finger in intervals that varied between 3–10 seconds. The total experiment time, including recording set up, was approximately 2.5 hrs.

### 4. EEG Data Analysis

#### 4.1 Data Preprocessing

The continuous EEG data were digitally filtered between 1∼120 Hz, and a 60 Hz notch filter was used to eliminate line noise. Trials with time-locked movement responses were extracted from filtered data. The data were divided into left thumb and right thumb conditions. The time period of a single trial was from 1000 ms before the response onset (the time point when the thumb pressed the response pad) to 1000 ms after response onset. Within each trial, bad channels were identified (defined as those with EEG>200 µV after smoothing with a moving average that is 80 ms long) and replaced using spherical spline interpolation. Epochs with artifacts due to eye blinks or muscle movements were detected and removed based on their typical signal characteristics and abnormal amplitude information (±25 µV). Only artifact-free epochs were retained for further analysis. The data were also re-referenced to the common average signal across all electrodes. Overall, the preprocessing procedure resulted in 164–197 trials per condition.

#### 4.2 ICA Denoising

After EEG preprocessing, independent component analysis (ICA) was performed in EEGLAB [33] with the extended Infomax algorithm using weight changes of 10^−7^ as a stop criterion to find the maximally temporally independent signals available. For each subject, the independent components (ICs) outside of the brain (primarily scalp or neck muscle activities), related to artifacts, and large residual variance were removed. The remaining independent components (ICs) were projected back to channel space then submitted to time-frequency (TF) analysis.

#### 4.3 Time-Frequency Analysis

Time-frequency analysis was performed on individual trials based on the Morlet wavelet analysis [34]. The time-frequency representation of power changes elicited by a movement was measured using a Gaussian window [35]. In this analysis, a total of 7 cycles was used to estimate the amplitude and phase of the signal. The wavelet's center frequencies range from 1 to 120 Hz in resolution of 1 Hz. The time window was moved in 10 ms increments, sliding across all time points. The power value of a single-trial was calculated for each time interval and frequency bin across all trials. Power changes (i.e., ERD and ERS, expressed in percentage change) were defined relative to the average of to the baseline interval (−1000∼−600 ms prior to the button press, where movement-related activity should be minimal, see Darvas et al. [15]) for each frequency. ERD and ERS were visualized after averaging across all trials. The frequency bands were defined as follows: theta  = 5–8 Hz, mu = 9–13 Hz, beta  = 14–30 Hz, low gamma  = 31–60 Hz, high gamma  = 61–120 Hz.

### 5. Head Modeling

Accurate estimates of cortical sources of scalp recorded voltage data required the construction of high-resolution electrical head models (Figure 1). High-resolution head models included accurate brain tissue segmentation, EEG sensor position registration, and specification of conductivity values for each tissue [4], [36], [37], [38], [39]. Additionally, dipoles work constrained to be perpendicular to the cortical surface. This has been shown to improve accuracy of source estimates [40].

We employed BrainK [41] to perform tissue segmentation from each participant's high resolution structural MRI data. Tissue segmentation classified each image voxel into the following tissue types: eyeball, scalp, skull, cerebral-spinal fluid (CSF), gray matter (GM), white matter (WM) and air. The WM and GM were further partitioned into the cortical surface and the cerebellum. The following conductivity values (in Siemens/meter) assigned to each tissue type are based on previously reported literature values: Eyeball  = 1.5, Scalp  = 0.44, Skull  = 0.018, (CSF)  = 1.79, (GM)  = 0.25, and (WM)  = 0.35 [36].

To specify dipole positions, the cortical surface was first characterized through the use of triangular meshes, which were parceled into patches of approximately equal size. All models used in the present study contained 1200 dipole patches per hemisphere. For each patch, perpendicular directions of vertices within the patch were averaged to derive the average, perpendicular orientation for that cortical patch. Once the head models were created, EEG sensor positions for each individual participant, derived from Geodesic Photogrammetry System (GPS) [42], were registered to the respective scalp surface. From the complete head model, a lead field matrix (LFM), which describes the propagation of current from each dipole position to each EEG sensor positions, was computed using the finite difference method (FDM) [43].

### 6. Cortical Source Estimation

Estimating neural sources of the frequency data involves the following steps: (a) wavelet transformation of the EEG data, (b) source estimation of the data in step (a), and (c) calculation of the ERD/ERS relative power changes in step (b). That is, a wavelet transform is first conducted to isolate the EEG frequency of interest for source localization, after which each frequency band of the continuous data set is transformed into source space through step (b). Finally, ERD and ERS metrics are computed for all dipole patches.

TF analysis was performed on individual trials using the following two equations:
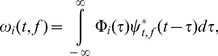
(1)


Where 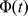 represents the scalp EEG data, 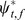 indicates the mother wavelet function, * is the complex conjugate, and 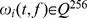 is the vector of wavelet coefficients for trial (i). Note that 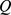 represents complex numbers.
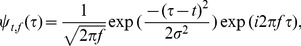
(2)


The Morlet wavelet function consists of a complex exponential multiplied by a Gaussian window. The trade-off ratio was set as 7 to create a wavelet family and 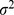 was 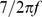. The tradeoff between spectral and time resolution was defined as a constant ratio, which is typically recommended to be greater than 5 in order to encompass at least one full sinusoidal cycle for any particular frequency [44]. The real and imaginary wavelet components, which represented the TF windows, were used for source estimation.

Estimation of cortical sources was performed using the standardized low resolution tomography (sLORETA) between scalp potentials and source amplitudes can be stated as the linear model:
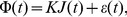
(3)where 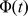 is the scalp potential measured at 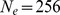 electrodes, 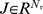
are the values of the distributed source dipoles, K is the lead field matrix (LFM), and 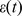 is the error term. Note that R represents real numbers. Many linear inverse methods can be obtained as the solution of the minimization problem:



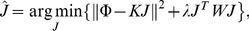
(4)

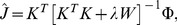
(5)where 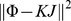 is the data fidelity term, and 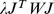 is the regularization term. 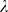 is the regularization weighting constant, *J* is the vector of source amplitudes (as defined above), and *W* defines the inverse technique (e.g., Minimum Norm, LORETA, LAURA etc.). In equation 4 (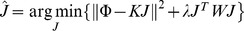), W in the present study is the identity matrix. Therefore, what is minimized in the entire 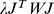 term is 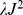, which is the *J* with minimum energy (i.e., the minimum norm).

The sLORETA method [45] is obtained by standardizing the minimum norm (MN) solution. In particular we may write 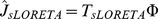 with the sLORETA source estimation matrix 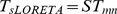, where 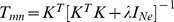 is the MN estimator,  is a diagonal matrix, and 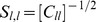.

sLORETA was applied to the complex-values derived from wavelet analysis and is expressed as
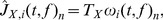
(6)where X is the sLORETA method applied to the complex value 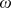 for a given source location (*n*) at time window *t* for a given trial (*i*) and frequency (*f*).

Additionally, the form of the analysis being described does not depend on *X* and it can be applied by other inverse solution methods.

Relative power changes (i.e., ERD/ERS) were calculated by comparing power at a particular time 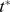 and frequency 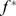 window to a baseline power computed from data in the −1000 to −600 ms window, as defined above in the Time-Frequency Analysis section above.

We first computed the squared absolute value of each point and subsequently average power of the dipole sources across trials, as 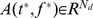


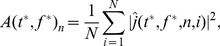
(7)where we employ the notation 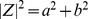 for a complex number 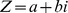.

Baseline power is computed using the source-localized Morlet wavelet coefficients corresponding to the same frequency 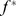, but at a time point chosen such the wavelet lies within the −1000 to −600 ms window. Due to the design of the wavelet filters, the length of the time window varies with 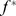 (higher frequency wavelets are supported on a small time window). We chose the baseline time 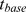 
such that the Morlet wavelet has the form (a, −600 ms). The baseline at frequency 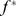 is then the vector 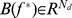 with components 
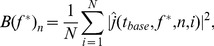
(8)We then define the (vector of) relative power differences at each dipole by



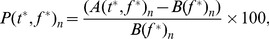
(9)This relative power changes 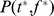 can be referred to as ERD or ERS, depending on whether it is negative or positive, and whether 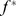 is in the HG band.

### 7. Evaluation of Source Estimates

In order to assess the ability to estimate the source generators of the observed ERD/ERS changes for individual participants, first we defined the regions of interest (ROIs) for the primary motor cortex (M1) and primary somatosensory cortex (S1) for each participant, see example in Figure 1(D). The hand region of M1 has been anatomically and functionally localized to an inverted omega-shaped knob (Ω) or a horizontal epsilon shape (viewed from axial slices) [46]. As noted by Yousry et al. [46] in intact brains (i.e., without structural abnormalities) and viewing the structural MRI data from the axial plane, high inter-rater reliability ratings were obtained for locating the hand-knob. In fact, in all subjects of the present study, the hand-knob was easily identified in the axial plane. Relative to functional localization of hand and finger movements to this region, we performed transcranial magnetic stimulation (TMS) for verification. The data were acquired for a different study and not presented here, but in all cases, TMS confirmed that finger movements were maximally activated in the identified M1 ROI.

Relative to the location of the hand-knob, the hand area of the S1 has been anatomically defined as a separate inverted omega (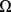) that resides ∼1–2 cm inferior to the hand-knob in the adjacent postcentral gyrus [47], [48]. In the present study, we identified the motor hand-knob landmark in an anatomical T1 MRI for all subjects and used all cortical dipoles patches residing in this location as our definition of M1. For S1, we selected the cortical dipole patches in a strip of the postcentral gyrus that runs 1–3 cm inferior to our definition of M1 and houses an inverted omega shape. The number of dipoles in combined M1 and S1 ROIs is between 23∼28 for each hemisphere for each subject. We selected a representative dipole from the combined M1/S1 ROIs that was on the central sulcus.

Next, five measures of source localization performance were calculated. The first measure was defined as the Euclidean distance from the representative dipole (anatomical position) to the peak and center of gravity (COG) positions of EEG and BOLD functional activity. This measure is calculated using the following formula:
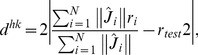
(10)where *r_test_* is the representative dipole, *r_i_* is the location of *i*th source, and 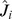 is the estimate of the dipole activity at location *r_i_*. The second measure is the distance of the EEG source location to the BOLD activity (peak to peak and COG to COG). The third measure is quantified as the ROI activity ratio (ROI-AR) and is defined as number of active dipoles in M1 and S1 to all dipoles within those ROIs. The fourth measure is defined as the ratio of the mean activity within the ROIs relative to the mean activity of the same hemisphere (ROI-SHR). The fifth measure is the ratio of the mean activity within the ROIs relative to the mean activity of both hemispheres (ROI-BHR). Standard error of the mean (SEM) is calculated for all measures.

### 8. MRI Data Acquisition

#### 8.1 Structural MRI

To derive anatomically accurate model of soft head tissues, T1-weighted scans using Siemen's MPRAGE sequence (repetition time (TR)  = 2.5 sec; echo time (TE)  = 3.4 ms; flip angle (FA)  = 8 degrees) with a 1×1×1 mm resolution covering 256 voxels in each spatial direction. Data were acquired in Siemen's 3T Skyra (subjects 1–4) or 3T Allegra (subject 5) scanners using 20-channel, head-neck coil and a quadrature birdcage head coil, respectively. Parallel imaging reconstruction was not performed.

Sequence times were 10 minutes 39 seconds on both systems. Foam padding was used to minimize head movements, and all subjects were highly cooperative and experienced with minimizing head motion over extended data collection periods. Good contrast and little to no artifacts were observed, with effective resolutions approaching the highest achievable for the sampling used. The head-only Allegra system images in the extreme inferior temporal regions (only) have lower contrast and there are localized blood flow artifacts (also in inferior regions), but these quality issues have little or no impact on either fMRI coregistration or tissue modeling.

#### 8.2 Tasks and fMRI Acquisition

The task structure is similar to the structure for the EEG recordings; participants were cued, with a letter near fixation, to move either the thumb or little finger forward and back about 2 cm (compared with pushing a response pad for EEG task), self-paced at about 2 Hz (compared with.5 Hz for EEG task). Finger movement blocks consisted of 3 to 10 second periods, interspersed with rest blocks (no movement). The total duration for each block type was approximately equal; for example, for each block type there were about 3.33 times as many 3-second blocks than 10-second blocks. The length and ordering of each block (thumb, little finger or rest) was a pseudorandom permutation of all block variations, with no two sequential blocks of the same type. For example, a thumb block of any length was followed by either a little finger or rest block of any length. The total duration of a single motor fMRI run was 5.5 minutes for a single hand. The experiment was repeated for the each hand.

Functional data were acquired with a 3T Siemens Allegra head only MRI system. Whole-brain gradient echo EPI images were obtained with the following parameters: TR = 2 seconds, TE = 30 ms, and FA = 80°. We collected 32 axial slices with a thickness of 3.125 mm with interleaved acquisition order. We used a Siemens' Prospective Acquisition Correction (PACE) protocol to compensate for head-motion in real time prior to acquisition of each whole brain image. No substantial motion was accounted for by PACE. In every fMRI run, the absolute compensation for displacement and rotational excursions across a session was less than 1.0 mm and 1.0 degrees, respectively. Inter-scan differences rarely exceeded 0.1 mm and 0.1 degrees.

### 9. fMRI Analysis

FMRI data processing was carried out using FEAT Version 5.98, part of FSL [49] (FMRI Expert Analysis Tool; FMRIB's Software Library, www.fmrib.ox.ac.uk/fsl). The following pre-statistics processing was applied; slice-timing correction; spatial smoothing using a Gaussian kernel of FWHM 4 mm; grand-mean intensity normalization; high-pass temporal filtering (cut-off = .0125 Hz), and residual motion correction. The task-related regressor was modeled as boxcars convolved with the FSL default canonical hemodynamic response function. Separate boxcars represented each digit movement and rest trial time courses. Time-series statistical analysis using the general linear model (GLM) was carried out using FMRIB's improved linear model (FILM) with local autocorrelation correction [50]. Functional images were coregistered to each individual participant's T1-weighted structural image with 6 degrees of freedom (translation and rotation only), which was then coregistered and resampled to the FSL standard brain (MNI/ICBM 152 template [51] using FMRIB's linear image registration tool (FLIRT) with 6 degrees of freedom [52]. Statistical maps were then resampled to 1×1×1 mm resolution for direct comparison to the structural image.

### 10. Registration of BOLD Data to Cortical Surface

Functional activation data from fMRI (BOLD) were mapped to the cortical surface used for dEEG source estimation (see above). To map the functional data to the dipole surface of the electrical head model, a transformation matrix was calculated that maps the fMRI volume into the space of the structural image used to generate the cortical surface. Each fMRI voxel was then related to a single dipole patch, with each dipole patch containing multiple fMRI voxels. The average number of voxels per dipole patch was 247. To determine the BOLD activity of each dipole patch, we first set a 2.5 Z-score threshold to statistically define an active voxel. Next, because there are multiple voxels within a dipole patch and in order to deal with outliers, we set a second threshold (“fractional threshold”) that specified the percentage of voxels in a dipole patch that must exceed the Z-score. We used a fractional threshold of 25%. For dipole patches that met these criteria, the voxels with activations over the Z-score threshold were then averaged to represent the activity of that dipole patch. The remaining dipole patches were set to zero.

## Results

**Figure 2:**
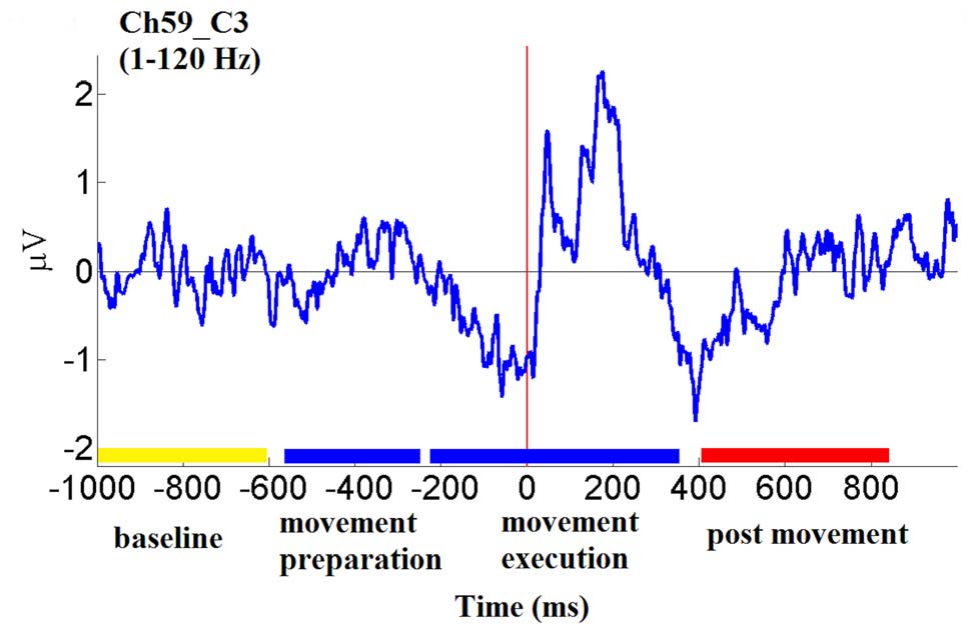
Time-course of average EEG trace (i.e., event-related potential) from channel C3 (bandpass: 1–120 Hz) during right thumb movement in one subject. Color bars on horizontal axis mark categorical time periods (see text).

Figure 2 presents the ERP recorded from an electrode over the contralateral motor cortex for a single subject. Familiar movement-related potentials can be observed in the movement execution period, particularly the negative going potential prior to the button press and positive peaks following the response [19]. Time-frequency analysis of individual trials that make up the ERP capture not only the frequency specific changes that are phase-locked to the response but also the non-phase locked features, providing a more detailed and comprehensive view of the brain changes associated with motor control. Although there are important motor-related low-frequency activities [53], low-frequency information requires a longer time interval. Because of the requirement for more data, it is difficult to obtain enough artifact-free trials at these longer intervals for analysis. Therefore, following time-frequency analyses are focused on frequency bands above 4 Hz.

**Figure 3:**
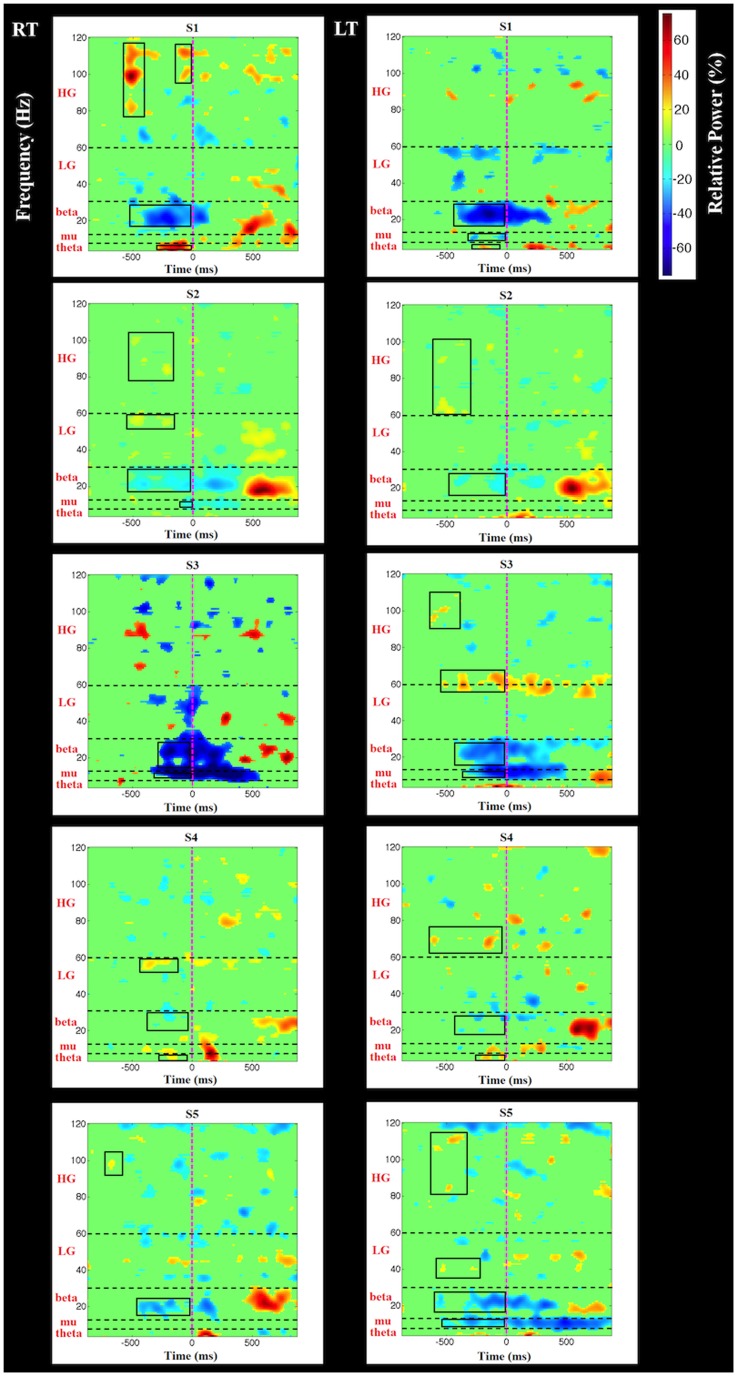
Subject specific time-frequency plots for left and right thumb movements. Data represent thresholded (top 10%) relative power changes (blue  =  decrease, red  =  increase, and light green  =  no significant change) at contralateral EEG channels (C3/C4). Time frequency maps aligned at time  = 0 ms (pink dashed line). Horizontal dash lines represent frequency band boundaries. Boxes represent ERD/ERS changes that are not artifactual (see text).

Contralateral motor area (channels C3/C4) time-frequency ERD/ERS maps for left and right thumb (RT/LT) movements are shown in Figure 3. We focused on the movement preparation and movement execution (up to the time of button press) periods. Movement-related ERD/ERS activities that were considered actual signals (i.e., not artifactual) were defined as those activities not restricted to just one channel but rather multiple contiguous channels (greater than 3, due to volume conduction). Only ERD/ERS activities that satisfied this requirement are highlighted in Figure 3 by boxes.

Starting with low frequency changes, pre-response theta-band ERS can be observed in two subjects. Unlike theta-band energy increases, mu-band energy changes were observed as an ERD, and this was present in four subjects (only subject 3 showed this response for both left and right thumb movements). The most robust spectral change is observed at the beta frequency; in all subjects for both response hands this was observed as an ERD. Unlike other spectral changes, beta-band ERD is sustained over hundreds of milliseconds and extends well into the post response period. The well known “beta rebound” (ERS) can be seen in all subjects and conditions, except subject 3 LT, and follows the termination of the beta band ERD (not highlighted in Figure 3). Low gamma-band activity was evident in three subjects and high gamma-band activity was present in all five subjects, although not in all conditions. Both low and high gamma-band activities are always seen as ERSs. Compared to lower frequency spectral changes, gamma-band ERSs are temporally discrete and can repeat several times before the button press (e.g., S1, S2, S4).

Table 1 shows the mean relative power changes (regardless of location) associated with left and right thumb movements at contralateral C3/C4 channels and S1/M1 source for each frequency. A paired t-test did not reveal any statistical differences between the overall power changes associated with left and right thumb movements.

**Table 1:** Average relative power changes for different frequency bands during left and right thumb movements for scalp (C3/C4) and source (M1 and primary somatosensory) data.

	Scalp (%)		Source (%)	
Frequency	RT	LT	RT	LT
Theta	43.64	23.71	50.38	33.76
Mu	37.57	23.16	46.73	30.47
Beta	29.64	29.12	39.99	39.76
LG	17.03	20.87	41.90	40.65
HG	37.67	25.71	80.27	56.77

### Localizations of Frequency Band Changes and BOLD Activation

**Figure 4:**
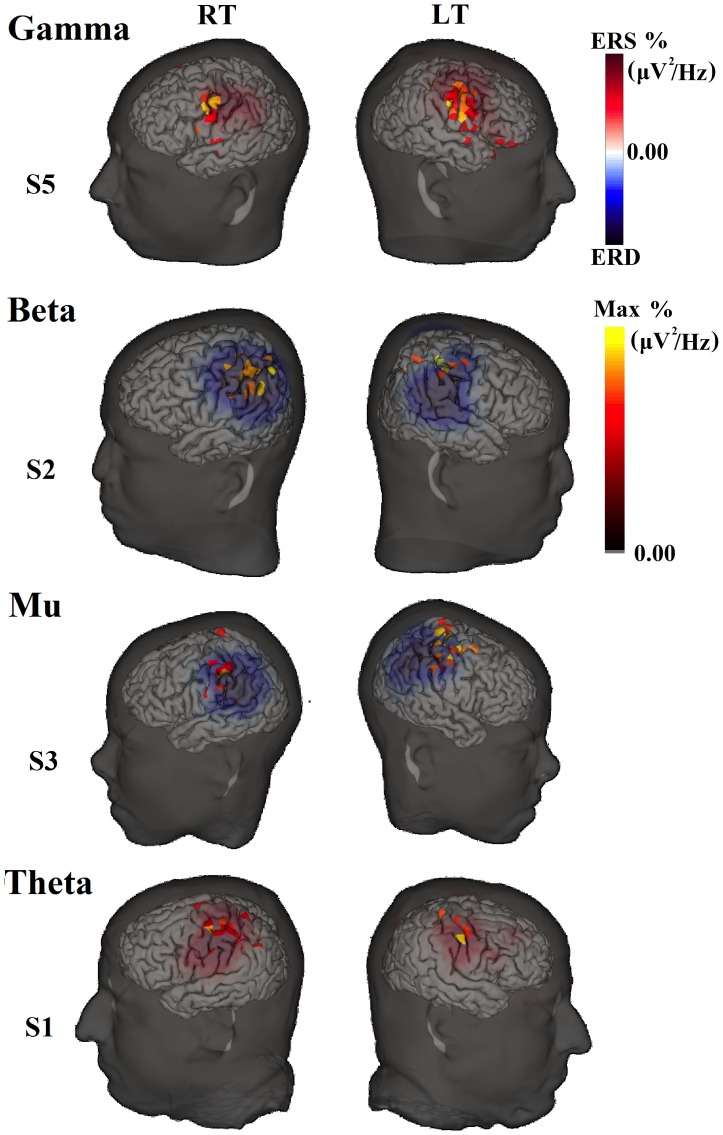
Movement-related spectral changes at the scalp and source for single subjects at four frequency bands. All scalp and source estimate results are thresholded at 95% and 97%, respectively. The scalp activities are indicated by either relative power decrease (ERD) or increase (ERS).

**Figure 5:**
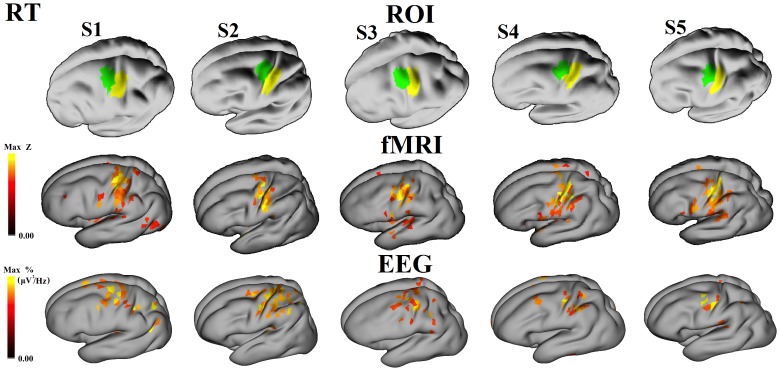
fMRI BOLD response and cortical distributions of beta-band premotor ERD during right thumb movements. Each column represents a different subject. The thresholds for the beta-band ERD were set 95% at scalp and 97% at source for each subject. The z score of BOLD activation was > = 2.5 and fraction 25% (see text). M1 and S1 ROIs are represented by green and yellow colors, respectively.

**Figure 6:**
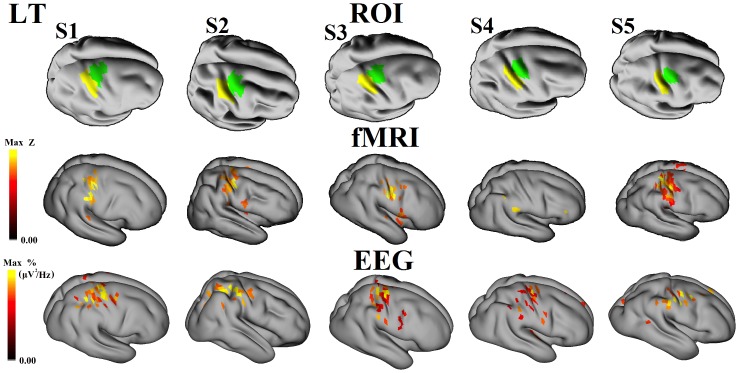
fMRI BOLD response and cortical distributions of beta-band premotor ERD during left thumb movements. Each column represents a different subject. The thresholds for the beta-band ERD were set 95% at scalp and 97% at source for each subject. The z score of BOLD activation was set at > = 2.5 and fraction 25% (see text).

Examples of sources associated with power changes in each frequency band are illustrated in Figure 4. ERD/ERS activities are shown at the scalp with the corresponding source estimates (on both wrinkled and inflated cortical surfaces, the latter showing activity in the sulci). Figures 5 and 6 show the localization results obtained with fMRI and beta-band ERD. In all 10 sessions (5 subjects and 2 conditions) for beta-band, activities are localized to the contralateral hemisphere at the anatomically estimated position of the hand representation in M1, to the adjacent primary somatosensory cortex, as well as extending slightly beyond these borders. The EEG source data represent activity that is equal to or greater than 97% of the maximum activity measured over all dipole patches. As noted in the Methods section, BOLD data at the cortical surface represent activity that are equal to or greater than a Z-score of 2.5 and 25% fractional threshold for each dipole patch.

**Figure 7:**
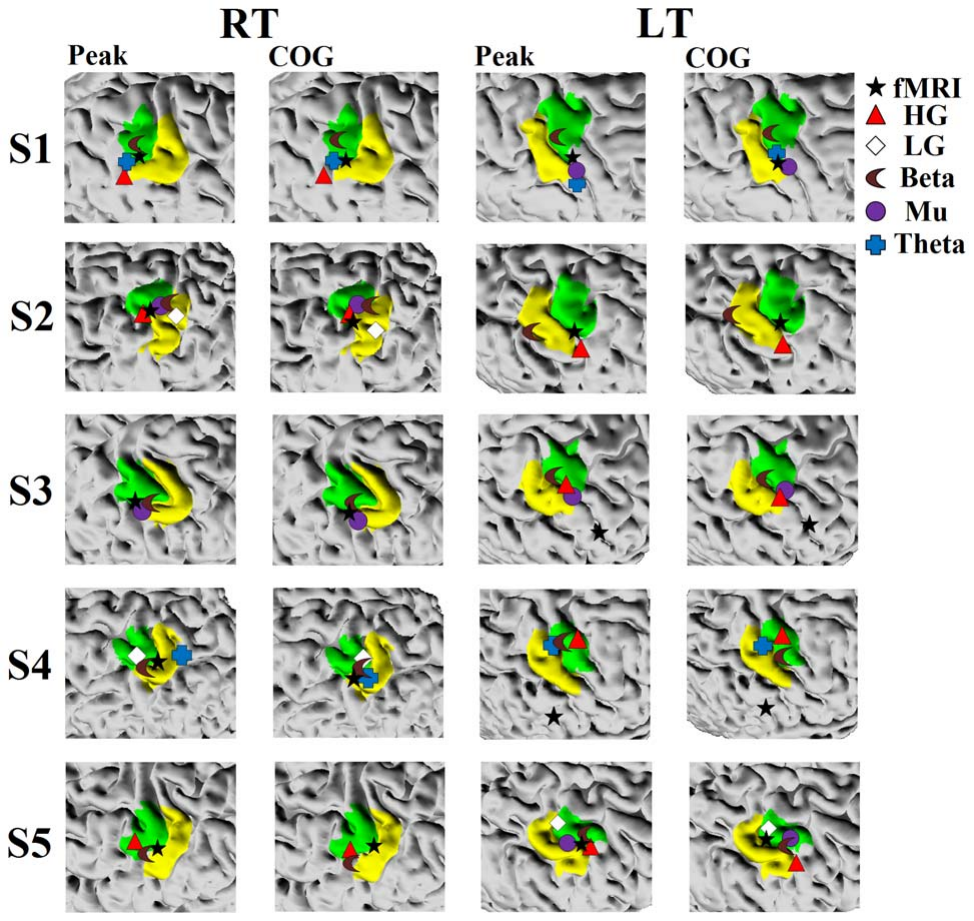
Magnified views of EEG and BOLD locations relative to M1 (green) and S1 (yellow) ROIs. The left hemisphere and right hemispheres are presented for RT and LT conditions, respectively.

Figure 7 summarizes the localization results for BOLD activation as well as source estimates for spectral band related changes. In this figure, both peak activation and COG locations are plotted for comparison. As can be seen, BOLD peak activation is located in M1 of both contralateral hemispheres in two subjects (S2 and S5) and one contralateral hemisphere in the other two subjects (S1 and S3). The peak location for S4 was just posterior to the M1 ROI for RT, but for LT it was clearly mislocalized according to the anatomical landmark. These results hold even when the COG measure is considered.

In S1, BOLD peak activity for the left thumb condition was just ventral to the M1 ROI. This is mainly due to the fact that there is strong activation of secondary somatosensory cortex for this condition (see Figure 6). For S3, the left thumb condition did not show much BOLD activation in the M1 ROI (see Figure 6). For S4, not only was BOLD activity in the M1 ROI lacking (particularly for the LT condition), it was also lacking in primary somatosensory cortex (see Figure 6). In fact, in S4, activity was generally not present in the pre and post central regions for LT. Therefore, we explored whether activity in M1 could be identified if we relaxed the threshold criteria. When the fractional threshold was set at 10% (with the Z-score threshold kept at 2.5) activity in the M1 ROI can be observed for S3 (although the peak of the activation remains unchanged as expected). In S4, activation in the M1 ROI is not observed, even at this threshold level.

In the EEG analysis, the frequency that consistently localized to the M1 ROI is beta-band. The exceptions were for the S2 left thumb condition for the peak measure, and S2 left thumb and S5 right thumb conditions for the COG measure. Although high gamma-band activity was present in most subjects (7 sessions out of 10), it was localizable (when peak location was considered) to M1 in only five sessions, and it was only localized to M1 in both hemispheres for S5. With COG as the metric, high gamma-band localization was in the primary somatosensory ROI for S3 and S5 (left thumb) rather than M1 ROI.

The remaining frequency bands showed much more variable localization. When both M1 and S1 are considered, however, the localizations of most frequency ERD/ERS activity are within these two ROIs. Particularly interesting is the comparison of the BOLD activation and ERD/ERS localizations in S3 and S4 for the left hand condition. For these subjects, localization of EEG activity appears to be more precise than that for BOLD, with the BOLD activity clearly centered outside and at some distance from either ROI.

### Localization Performance

Table 2 presents two distance measures. The first measure quantifies (across all subjects) the mean distance from the central sulcus to the peak and COG positions of BOLD and frequency specific ERD/ERS. This measure standardizes the distance metric to an anatomical position that allows for comparison between EEG and BOLD localization, and has been used by others for distance calculation in the evaluation of source localization accuracy for S1 activity [54]. The second measure quantifies the distance of the EEG sources to the BOLD activity (using peak-to-peak and COG-to-COG). To put the distance measurement in perspective, the average distance from the center of one dipole patch to a neighboring dipole patch is approximately 7 mm.

**Table 2:** Mean distance (±SEM). Anatomical position is distance relative to central sulcus.

	Anatomical position (mm)		Distance from fMRI (mm)	
	Peak	COG	Peak	COG
BOLD	19.831 (±6.47)	18.211 (±5.80)		
Theta ERS	18.691 (±6.91)	14.776 (±5.81)	12.178 (±1.47)	11.670 (±3.05)
Mu ERD	9.927 (±4.99)	16.407 (±2.87)	14.718 (±4.11)	15.363 (±1.86)
Beta ERD	15.394 (±4.85)	11.978 (±5.11)	23.636 (±5.16)	19.725 (±3.60)
LG ERS	19.778 (±6.08)	24.123 (±9.49)	26.950 (±8.07)	22.008 (±9.18)
HG ERS	17.436 (±3.92)	21.780 (±2.66)	19.955 (±4.53)	23.230 (±5.35)

The mean distance calculations are across subjects.

Relative to the central sulcus, the peak BOLD position is largest of all the measures, due to the fact that in the S4 left thumb session, noted above, peak activity was not observed in M1 or S1. When this session was excluded from the distance calculation, the mean BOLD peak and COG were reduced to 14.686 mm (±4.38) and 12.824 mm (±2.31), comparable to the EEG measures. Although mu-band activity has the lowest peak distance measure, it was only seen in five out of ten sessions, and the difference between the distance measure for mu-band activity and the next smallest distance (beta-band) is about one dipole patch position. When the outlier session for the beta-band (S2 LT) localization was removed, peak distance measure was reduced to 10.792 (±1.73), comparable to the mu-band localization but with lower standard error. Theta-, LG-, and HG-band locations are approximately equivalent. When the COG measure is considered, beta-band activity shows the smallest distance measure, even when the outlier was not removed (7.596 (±2.94), with outlier removed).

Relative to the BOLD positions, theta-band activity showed the closest correspondence to BOLD. The beta-band results in Table 2 include the outlier session (S2 LT). When this session was removed from the analysis, the Peak and COG metrics were reduced to 19.492 (±3.03) and 16.876 (±2.49).

To compare the cortical spatial extent of BOLD and EEG frequency-band changes, three metrics were calculated: 1) ROI-AR, 2) ROI-SHR, and 3) ROI-BHR. These performance metrics are highest for the beta and mu EEG measures. However, these EEG measures were still not as responsive and discriminating as the BOLD measure, even when S4 left thumb session is included, as in Table 3. When we exclude S4's left thumb session from the BOLD ROI-AR and ROI-SHR/ROI-BHR metrics, these values increased to 0.504 (±0.07) in ROI-AR, 15.460 (±3.81) in ROI-SHR and 20.815 (±6.23) in ROI-BHR.

**Table 3:** Performance Metrics.

	ROI-AR	ROI-SHR	ROI-BHR
BOLD	0.450(±0.09)	15.317(±4.61)	20.565(±7.77)
Theta ERS	0.214(±0.03)	9.238(±3.06)	8.080(±1.31)
Mu ERD	0.341(±0.06)	10.045(±2.27)	13.150(±2.21)
Beta ERD	0.306(±0.05)	9.580(±1.83)	10.969(±2.32)
LG ERS	0.184(±0.06)	6.191(±3.78)	7.161(±2.00)
HG ERS	0.161(±0.04)	6.386(±2.03)	6.736(±2.09)

The threshold for EEG cortical distributions is set 97% at source for each subject. The z score of BOLD activation are calculated using thresholds 2.5 and fraction 25% (see text for details). Data for these three metrics represent the average across all subjects.

## Discussion

In the present study, we examined the spatial resolution of movement-related spectral changes in the 256-channel dense-array EEG (dEEG) in multiple frequency bands using source localization to the individual's cortical surface with high-resolution individual head models. These effects were examined in the movement preparation and movement execution periods before the button press and were compared to movement-related BOLD changes in the same individuals. Although the BOLD analysis characterized movement-related activity, the lack of temporal resolution precludes separation of pre-movement from post-movement activity.

Although the BOLD results of course varied with the degree of thresholding of the effect, the BOLD measure showed greater ROI-AR (more activity in the ROI) and ROI-SHR/ROI-BHR metrics (less activity outside the ROI) than any of the EEG measures. There were consistent BOLD effects in the postcentral (somatosensory) cortex as well, but given the lack of temporal resolution, we could not discern whether these were related to the movement preparation or the somatosensory response to the button press.

EEG effects were observed across a wide frequency band and could be isolated to the pre-movement and movement intervals. The EEG effects were observed in all subjects and conditions, and it was the beta-band desynchronization (ERD) that showed the most robust effect. This finding is similar to the observation by Seeber et al. [55], using 120 EEG channels in a walking task and a boundary element head model, that beta-band activity was more consistent compared to mu-band activity, localized to the medial aspect of the sensorimotor cortex. Although the beta ERD was not highly focal in the present study, it was centered on the hand area consistently. When peak activity was examined, beta-band sources were localized to the hand region of M1 in 9 out of 10 sessions. When COG was used as the metric, beta-band activity was localized to the M1 in 8 out of 10 sessions.

The localization of beta-band ERD to the hand knob region of M1 in the present study is consistent with magnetoencephalographic (MEG) findings that show 20-Hz, finger movement-related MEG oscillations to be localizable to the hand knob region [56], [57]. Of the studies that have examined beta-band ERD, the research performed by Yuan et al. [31] is the most comparable to our study. These authors used parallel EEG (62-channel) and fMRI studies to localize finger and foot movement-related activations. Similar to our study, the fMRI activation was located in the M1 hand region for hand movements. Beta-band activity spanned both the pre- and postcentral gyri at the level of the hand representation, but peak location was in the postcentral gyrus. The difference in beta-band localization between the present study and those obtained by Yuan et al. [31] may be due to EEG channel count difference as well as the resolution of the head models employed. In this study used many more EEG channels as well as higher resolution head models.

The frequency that showed the second most robust response was the high gamma-band. It was present in 7 out of 10 sessions and was located in M1 in 5 sessions. Ball et al. [16] showed, using intracranial EEG recordings, that high gamma-band activity was predominantly localized to the hand and arm representation region in M1. These researchers observed high gamma-band activity along the midline which we did not observe. This could be due to the fact that they had a much more complicated self-paced motor task that likely required more planning, which would be expected to engage the motor planning regions of the medial prefrontal cortex, such as the SMA. Low gamma-band activity was seen uniquely (i.e., not in the presence of high-gamma band activity) in only one (S4, RT) of the three sessions, and it was localizable to M1 in one session. Localization results for alpha- and theta-band activities were variable relative to the hand area (M1 ROI). As noted, Seeber et al. [55] found localization of alpha-band to be somewhat variable. For theta-related movement potentials, Luu and Tucker [20] found that regions spanning the primary motor and primary somatosensory cortices were source generators of the scalp potentials.

An important finding was that for both BOLD and EEG measures, the movement-related activity was typically observed in both primary motor and somatosensory areas, similar to the observations by Yuan et al. [31]. Additionally, activity was also observed in contralateral premotor, secondary somatosensory, and parietal cortices. Although activation of multiple regions observed in the BOLD data could be attributed to the poor temporal resolution between pre- and post-response intervals, this pattern was also observed with EEG data, where the data analysis clearly focused on activity prior to the button press. The non-focal nature of the EEG source solution could be due to poor spatial resolution, including the impact of different tissue resistivities to current flow (which causes smearing), the sparse sampling (∼2 cm intersensor distance) of the scalp potential field, and the ill-posed nature of the inverse problem. Whereas these imprecisions are clearly important, we think that the activities in both somatosensory and motor cortices are indeed characteristic of the neurophysiology of cortical action control for several reasons.

First, it has been shown structurally that, unlike the structural isolation of other primary sensory areas from each other and from primary motor cortex, primary motor and somatosensory areas share direct u-shaped cortical connections [58]. Second, examination of single unit responses in M1 and primary somatosensory cortex show functional inter-leaving of motor and cutaneous responses, particularly within the central sulcus [59]. Finally, direct recordings from the cortical surface in humans show that motor-related beta-band ERDs are recorded by local electrodes over both primary motor and somatosensory cortices [60].

Although BOLD responses spanned both somatosensory and motor cortex, the peak activation or COG metrics were located in M1 or just posterior to M1 (with the exception of S3 and S4 LT) but never in primary somatosensory cortex. Similarly, although beta band-activity covered a wider area than M1, peak and COG metrics also were always located primarily to M1 (with exception of S2 LT), in contrast to all other frequency changes. This finding is remarkably similar to those reported by Miller et al. [60], who compared beta- and theta-band localization (recorded with intra-cranial electrodes) relative to fMRI, and found that it was beta-band activity for thumb movements that overlapped the most with BOLD activation.

When both peak and COG measures of localization accuracy were measured in relation to the central sulcus at the level of M1 and primary somatosensory cortex (as done by Bai et al. [54]), the beta-band ERD was the most accurate indicator (when the S2 LT outlier was removed). BOLD localization showed the second best performance (when S4 LT session was excluded). Mu-band localization was equivalent to beta-band localization only for the peak measure. Localization results showed that the remaining frequency bands were less likely to be located in M1.

When the peak and COG locations of the BOLD response were used as the target location measures, theta-band activity is closest, followed by mu- and beta-band activity. Low and high gamma-band activities had the least consistent relation to the BOLD locations.

With respect to ROI-AR, ROI-SHR, and ROI-BHR performance metrics, the BOLD response was the superior measure. Of the EEG measures, when considering these metrics, mu-band and beta-band changes showed the best discrimination of location, regardless of the employed threshold (see Table 3). For the several reasons of spatial imprecision noted above, including the limited number of electrodes over the motor area, the EEG localization is expected to be technically inferior to the BOLD measure. However, it could also be that the EEG changes are indeed less focal than the hemodynamic response, reflecting the nature of inter-regional communication processes indexed by oscillatory activity.

In this respect, it may be important that Miller et al. [60] recently reported with intracranial (subdural) EEG that broadband, non-oscillatory EEG activity is sufficiently focal to discriminate individual finger movements. Interestingly, the broadband changes thought to index unsynchronized neuronal firing were entrained to the phase of beta-band oscillations, and these beta oscillations were not focal but were synchronized over larger regions than just the active finger. It will be important in future research to determine whether such broadband changes, which have only been reported for intracranial recordings, can be observed with scalp (head surface) EEG, and whether they demonstrate the precision of the intracranial broadband data.

### Limitations of the Present Study

Even though we defined the ROIs based on well-known, easy to identify, and validated landmarks of the primary hand region [46], this is still a manual process. Our definitions of the two ROIs are likely larger than the actual functional M1 and S1 hand areas, but they will not affect the primary performance measures as the ROIs are applied equally to all ERD/ERS and BOLD localizations. Moreover, we tried to mitigate this manual ROI definition through the use of the central sulcus for the distance comparison, as others have done [56].

A second limitation is that the resolution of the localization for both EEG and BOLD measures is limited by the size of our dipole patches. Because BOLD activation, which is at the voxel resolution, was grouped into dipole patches, the resolution of BOLD reported in the present study is not optimal. However, this approach did allow us easily register the BOLD data to the same space as the EEG source data, allowing direct comparisons in relation to the individual's cortex. Third, some metrics, such as ROI-AR and ROI-SHR/ROI-BHR, are dependent on the threshold employed for both EEG and BOLD data. However, even with this dependency, it does appear that BOLD results are not only more sensitive but more specific than EEG localization with the present methods.

A third limitation is our choice for BOLD statistical thresholds. We did not use standard procedures for setting single-subject statistical thresholds while accounting for multiple comparisons. We did examine standard statistical parametric maps that account for multiple comparisons, and the results revealed variable statistical power between subjects. However, the results obtained with this standard approach hid, to some degree, the commonality of responses across subjects that we wished to examine with our threshold choices.

Finally, in the present study, we only focused on the localization of oscillatory activity, but it is possible that other EEG signals that have been shown to be much more focal, such as broadband activity noted above, could be much more sensitive and specific.

### Conclusions

In studying the localization of movement-related ERD/ERS of the EEG using high-resolution head models, in comparison to BOLD localization, we found that across all frequency bands, beta-band ERD was the most robust response (being present in all subjects and conditions) and was consistently centered on the hand area. Beta-band ERD had the lowest localization distance to the central sulcus, even when compared to the BOLD measure. Beta ERD was similar to BOLD in being consistently focused on M1. BOLD responses were always more sensitive and specific in relation to focal activations than any of the EEG measures. However, in two sessions, the BOLD response was completely lacking in primary motor cortex or the peak activity was at some distance from M1 of the hand region. In those sessions, beta-band ERD was observable in M1.
