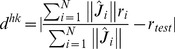# Correction: Localizing Movement-Related Primary Sensorimotor Cortices with Multi-Band EEG Frequency Changes and Functional MRI

**DOI:** 10.1371/journal.pone.0116433

**Published:** 2014-12-19

**Authors:** The PLOS ONE Staff

Equation 10 is incorrect. Please view the correct Equation 10 here.